# Surface Modification of Biodegradable Microparticles with the Novel Host-Derived Immunostimulant CPDI-02 Significantly Increases Short-Term and Long-Term Mucosal and Systemic Antibodies against Encapsulated Protein Antigen in Young Naïve Mice after Respiratory Immunization

**DOI:** 10.3390/pharmaceutics14091843

**Published:** 2022-09-01

**Authors:** Jacob E. Parriott, Jason P. Stewart, D. David Smith, Stephen M. Curran, Christopher D. Bauer, Todd A. Wyatt, Joy A. Phillips, Elizabeth Lyden, Geoffrey M. Thiele, Joseph A. Vetro

**Affiliations:** 1 Department of Pharmaceutical Sciences, College of Pharmacy, 986020 University of Nebraska Medical Center, Omaha, NE 68198, USA; 2 Department of Biomedical Sciences, Creighton University, 2500 California Plaza, Omaha, NE 68178, USA; 3 Research Service, Department of Veterans Affairs Omaha-Western Iowa Health Care System, Omaha, NE 68105, USA; 4 Pulmonary, Critical Care and Sleep, Department of Internal Medicine, University of Nebraska Medical Center, Omaha, NE 68198, USA; 5 Department of Environmental, Agricultural and Occupational Health, University of Nebraska Medical Center, Omaha, NE 68198, USA; 6 Donald P. Shiley BioScience Center, San Diego State University, San Diego, CA 92182, USA; 7 Department of Biostatistics, College of Public Health, University of Nebraska Medical Center, Omaha, NE 68198, USA; 8 Department of Internal Medicine, Division of Rheumatology & Immunology, University of Nebraska Medical Center, Omaha, NE 68198, USA; 9 Center for Drug Delivery and Nanomedicine, 985830 University of Nebraska Medical Center, Omaha, NE 68198, USA

**Keywords:** mucosal immunization, mucosal vaccine, vaccine delivery, administration volume, targeted vaccines, M cell targeting, dendritic cell targeting, C5aR1, C5a1R, CD88, EP54, EP67

## Abstract

Generating long-lived mucosal and systemic antibodies through respiratory immunization with protective antigens encapsulated in nanoscale biodegradable particles could potentially decrease or eliminate the incidence of many infectious diseases, but requires the incorporation of a suitable mucosal immunostimulant. We previously found that respiratory immunization with a model protein antigen (LPS-free OVA) encapsulated in PLGA 50:50 nanoparticles (~380 nm diameter) surface-modified with complement peptide-derived immunostimulant 02 (CPDI-02; formerly EP67) through 2 kDa PEG linkers increases mucosal and systemic OVA-specific memory T-cells with long-lived surface phenotypes in young, naïve female C57BL/6 mice. Here, we determined if respiratory immunization with LPS-free OVA encapsulated in similar PLGA 50:50 microparticles (~1 μm diameter) surface-modified with CPDI-02 (CPDI-02-MP) increases long-term OVA-specific mucosal and systemic antibodies. We found that, compared to MP surface-modified with inactive, scrambled scCPDI-02 (scCPDI-02-MP), intranasal administration of CPDI-02-MP in 50 μL sterile PBS greatly increased titers of short-term (14 days post-immunization) and long-term (90 days post-immunization) antibodies against encapsulated LPS-free OVA in nasal lavage fluids, bronchoalveolar lavage fluids, and sera of young, naïve female C57BL/6 mice with minimal lung inflammation. Thus, surface modification of ~1 μm biodegradable microparticles with CPDI-02 is likely to increase long-term mucosal and systemic antibodies against encapsulated protein antigen after respiratory and possibly other routes of mucosal immunization.

## 1. Introduction

The primary requirement for an effective vaccine is the ability to safely generate long-term protective adaptive immune responses against the targeted pathogen above threshold levels that correlate with a significant decrease or elimination of pathogen-related infectious disease [1,2,3,4]. Most licensed vaccines protect against infectious diseases by generating sufficient levels of long-term systemic antibodies after IM or SQ administration [5,6,7] that protect against invasive infections and likely provide backup protection against infections in the lower respiratory tract [8,9]. Administering vaccines by a mucosal route (i.e., oral, nasal, sublingual, buccal, pulmonary, rectal, or vaginal) may provide several advantages over systemic vaccines, including (i.) generating both mucosal and systemic antibodies to protect against initial infection at the portal of entry for most pathogens as well as subsequent invasive infection, (ii.) vaccine immunogenicity regardless of pre-existing systemic immunity, (iii.) the option for frequent boosting, (iv.) easy, pain-free administration that requires little training and increases patient compliance without the risk of spreading blood-borne infections, and (iv.) lower production costs and regulatory burden compared to systemic vaccines [9,10,11,12,13,14].

Currently licensed mucosal vaccines (8 oral and 1 intranasal) are composed of live, live attenuated, or inactivated strains of pathogens that are the most likely to generate the appropriate long-lived protective mucosal and systemic antibodies [9]. These vaccine types, however, are (i.) limited to pathogens that increase protection after natural infection and can be grown in culture, (ii.) are difficult to establish for most bacterial pathogens, (iii.) take a long time to develop, (iv.) are rarely safe and stable, (v.) may not cross-protect against other pathogenic strains, and (vi.), in the case of live/live attenuated vaccines, are not suitable for pregnant women or immunocompromised patients and have the remote possibility of reverting to wild-type virulence [10,15,16,17]. 

One approach to potentially overcoming the limitations of currently licensed mucosal vaccines is through mucosal administration of one or more protective antigens (i.e., subunit and recombinant vaccines) [15,18,19] encapsulated in nanoscale biodegradable particles. This can decrease mucosal vaccine degradation and clearance, increase localization to mucosa-associated lymphoid tissues (MALT) (major induction sites of adaptive immune responses), increase the levels and duration of epitope presentation and cross-presentation after internalization by antigen-presenting cells (APC), and increase the magnitudes of short-lived mucosal and systemic adaptive immune responses following mucosal administration [20,21,22,23,24,25,26,27,28,29,30]. Recombinant vaccines can also be designed to generate more potent and broadly protective memory B-cells and T-cells [31]. Encapsulated and unencapsulated subunit and recombinant vaccines, however, require the incorporation of a suitable mucosal immunostimulant to sufficiently activate APC (especially dendritic cells) and generate high levels of long-lived mucosal and systemic adaptive immune responses [32].

Cholera toxin subunit B (CTB) is the only mucosal immunostimulant incorporated as part of a licensed mucosal vaccine (Dukoral: oral, inactivated vaccine) [33,34] but is unsafe for IN administration [35,36] and possibly other routes of mucosal immunization. The most widely developed preclinical immunostimulants are based on pathogen-associated molecular patterns (PAMPs) [37,38,39]. Development and/or incorporation of PAMP-based immunostimulants, however, is extremely challenging due to the large number of PAMP receptors, differences in PAMP receptor activities/cellular distributions, differences in adaptive immune responses and levels of inflammation by individual PAMPs, the complexity and expense of PAMP molecules, and difficulties establishing stable formulations [10,13,40]. Thus, there continues to be a great need for the preclinical development of mucosal immunostimulants that are sufficiently potent, minimally pro-inflammatory, and safe for mass immunization [9,10,12,41].

In contrast to pathogen-derived PAMPs, we previously developed complement peptide-derived immunostimulant-02 (CPDI-02) (formerly “EP67”) as a novel second-generation, host-derived decapeptide agonist of C5a receptor 1 (hC5aR1/hC5a_1_R/CD88) [42,43] based on the C-terminal pharmacophore of human C5a (hC5a) ligand that acts as a mucosal and systemic immunostimulant [43,44,45] and adjuvant [42,46,47,48,49]. CPDI-02, unlike hC5a, selectively activates primary human mononuclear phagocytes (monocytes, monocyte-derived macrophages, and monocyte-derived DC) with ~1000-fold higher potency than primary human neutrophils (NP) (167 nM EC_50_ in MP vs. 160 μM EC_50_ in NP) (“MP-selective activation”) [50]. Directly conjugating CPDI-02 to peptide epitopes, whole proteins, or live pathogens increases the magnitudes of humoral and cellular immune responses in mice after mucosal or systemic immunization while minimizing potential neutrophil-mediated toxicity [42,44,46,47,48,49,51]. Combining CPDI-02 with CpG and Montanide also increases the magnitude and quality of B-cell and T-cell adaptive immune responses against a protective APol-solubilized membrane protein after systemic/respiratory immunization and subsequent protection of naïve mice against primary respiratory challenge with *C. trachomatis* [52].

Lower respiratory infections are the fourth leading cause of death worldwide [9] and develop primarily from initial infections of the upper respiratory tract [53]. As such, developing mucosal vaccines for respiratory immunization that can increase long-term protective mucosal antibodies in the upper and lower respiratory tract in addition to protective systemic antibodies could significantly decrease the incidence of infectious disease. 

We recently found that surface modification of biodegradable nanoparticles (~380 nm) with ~0.1 wt% CPDI-02 through 2 kDa PEG linkers (i.) increases the activation of immature murine bone marrow-derived DC (BMDC) and (ii.) increases long-lived memory subsets of CD4^+^ and CD8^+^ T-cells against an encapsulated model protein immunogen, LPS-free ovalbumin (OVA), in the lungs and spleens of young naïve female C57BL/6 mice after respiratory immunization and subsequent protection against respiratory challenge with OVA-expressing *L. monocytogenes* [3]. Given that the activation of dendritic cells (DC) is required to increase the generation of both long-lived memory B-cells and T-cells [54], we hypothesized that surface modification of biodegradable microparticles with CPDI-02 will increase the generation of long-term mucosal and systemic antibodies against encapsulated protein antigen after respiratory immunization. To test this hypothesis, we encapsulated LPS-free OVA in biodegradable PLGA 50:50 microparticles (MP) (~1 μm diameter) alone or MP surface-modified with inactive scrambled scCPDI-02 (scCPDI-02-MP) or CPDI-02 through 2 kDa PEG linkers (CPDI-02-MP) at ~0.4 wt%. We then compared the extent to which intranasal administration of CPDI-02-MP or scCPDI-02-MP increases (i.) magnitudes of short-term OVA-specific antibody-secreting cells (ASCs) in the lungs and spleen 6 days post-immunization, (ii.) titers of short-term (14 days post-immunization) and long-term (90 days post-immunization) OVA-specific antibodies in the nasal cavity, lungs, and sera, and (iii.) long-term signs of inflammation in the lungs 90 days post-immunization compared to vehicle alone.

## 2. Materials and Methods

### 2.1. LPS Removal from Ovalbumin (OVA)

LPS endotoxin was removed from Grade V hen egg white ovalbumin (OVA: 385 amino acids, MW: 44,287 Da, Sigma) [40 mg] using a Detoxi-Gel™ column (Thermo Scientific) as we previously described [3]. LPS-free ovalbumin (“OVA”) was used in all microparticle formulations and assays.

### 2.2. Synthesis of CGRR-CPDI-02 and CGRR-scCPDI-02 Peptides

CPDI-02 (YSFKDMP[MeL]aR where “MeL” = N-methyl leucine and “a” = D-alanine; formerly “EP67”) or inactive scrambled scCPDI-02 ([MeL]RMYKPaFDS; formerly scEP67) were activated with sulfhydryl groups by introducing N-terminal cysteine during solid-phase synthesis through a cleavable glycine double arginine linker (GRR). The resulting 14-amino acid peptides (CGRR-CPDI-02 and CGRR-scCPDI-02) were purified and characterized as described [50].

### 2.3. Encapsulation of OVA in Biodegradable Microparticles Surface-Modified with CPDI-02

OVA was encapsulated into biodegradable PLGA 50:50 microparticles (MP) (~1 µm in diameter) by the emulsion solvent evaporation method (ESE) at a theoretical loading of 10 wt% (mass OVA/mass of formulation) by the emulsification solvent extraction method (ESE). MP were surface modified with Cys-CPDI-02 or inactive Cys-scCPDI-02 through 2 kDa PEG linkers during OVA encapsulation by interfacial activity-assisted surface functionalization (IAASF) as we previously described [3] with modifications including using higher MW ester-terminated poly d,l-lactic-co-glycolic acid (PLGA 50:50; research grade; inherent viscosity 0.65 dL/g; Lactel Pelham, AL) [50 mg PLGA/mL; 2 mL], sonicating (UP200ST ultrasonic homogenizer with an S26d14 sonotrode, Hielscher Ultrasound Technology) the primary water-oil emulsion (W_1_/O) at full amplitude for 2 min, forming a crude secondary water-oil-water emulsion (W_1_/O/W_2_) by vortexing [400 RPM] for 25 s, and sonicating the crude secondary W_1_/O/W_2_ emulsion with a total energy of 60 Ws (60 J) at full amplitude.

### 2.4. Quantitation of OVA Loading in MP by Ultra-Performance Liquid Chromatography (UPLC)

MP [2 mg] were equilibrated to r.t., dissolved in DMSO [125 μL] in an 8-mL borosilicate glass vial, and incubated (r.t., 1 h) with constant shaking. A digestion solution (NaOH [0.05 M]/SDS [0.5% *w*/*w*] in deionized H_2_O) [1.25 mL] was added to the MP and the entire solution was stirred [650 RPM] in a capped vial overnight. Undissolved polymer was pelleted [10,000 RCF, r.t., 10 min] and the supernatant was transferred to new 8-mL vial where 10% trifluoroacetic acid (TFA) in dH_2_O [62.5 μL] was added to maintain pH compatibility with the UPLC column (pH 2 to 12). OVA standards were prepared by treating a concentrated stock solution in deionized H_2_O under the same MP digestion conditions then serially diluting in digestion solution. MP samples and OVA standard concentrations were determined by UPLC (ACQUITY UPLC H-Class PLUS System, Waters, Milford, MA, USA) using a reversed-phase C4 column (ACQUITY UPLC Protein BEH C4 Column, 300A, 1.7 μm, 2.1 × 50 mm, Waters) with Solvent A (0.1% TFA (*v*/*v*) in deionized H_2_O) and Solvent B (0.1% TFA (*v*/*v*) in acetonitrile). OVA was eluted from the column by increasing the percentage of solvent B from 0 to 100% over 20 min with continuous monitoring of column effluent at 214 nm. A linear equation for the standard curve of OVA concentration (*Y*-axis) vs. OVA peak AUC (*X*-axis) (y = mx + b) was calculated by linear regression to determine sample OVA concentration from sample AUC where x = sample AUC and y = sample OVA concentration. Average wt% OVA loading ± propagated SD (*n* = 3 replicates from at least two independent batches) was calculated as follows:

$$\mathrm{OVA}\;\mathrm{loading}\;\left(\mathrm{wt}\%\right)=\frac{\mathrm{Sample}\;\mathrm{OVA}\left[\frac{\mathrm{mg}}{\mathrm{mL}}\right]\;\times \;\mathrm{Sample}\;\mathrm{Volume}\;\left[1.4375\mathrm{mL}\right]}{\mathrm{Mass}\;\mathrm{of}\;\mathrm{Sample}\;\mathrm{MP}\;\left[2\mathrm{mg}\right]}\times \;100$$

and percent encapsulation efficiency (EE%) was calculated as follows:

$${\mathrm{EE}\%}_{\mathrm{OVA}}=\frac{\mathrm{Sample}\;\mathrm{mg}\;\mathrm{OVA}/\mathrm{mg}\;\mathrm{MP}}{\mathrm{Theoretical}\;\mathrm{mg}\;\mathrm{OVA}/\mathrm{mg}\;\mathrm{MP}}\times \;100$$

### 2.5. Quantitation of OVA Burst Release from MP

Burst release of OVA (average percent of total OVA released 24 h after resuspension of lyophilized MP) was determined as we previously described with modification [3]. MP [10 mg] were suspended in PBST (PBS and Tween-20 [0.05% *v*/*v*]) [1 mL], vortexed [20 s], and incubated [37 °C] with shaking (Vortemp 56 Shaking Incubator) [200 rpm/min] for 24 h. Samples were pelleted [10,000 RCF, 5 min], supernatants stored at −20 °C, and concentrations of OVA in supernatants determined by UPLC (Section 2.4). Average % OVA burst release ±SD (*n* = 3 replicates from at least two independent batches) was calculated as follows:

$${\mathrm{Burst}\;\mathrm{release}}_{\mathrm{OVA}}=\left[\frac{\mathrm{Sample}\;\mathrm{OVA}\;{\left[\mathrm{mg}/\mathrm{mL}\right]}_{\mathrm{supernatant}}\;\times \;\mathrm{Sample}\;\mathrm{Volume}\;\left[1\;\mathrm{mL}\right]}{\frac{\mathrm{Sample}\;\mathrm{OVA}\;\left[\mathrm{mg}\right]}{\mathrm{mass}\;\mathrm{of}\;\mathrm{particles}\;\left[\mathrm{mg}\right]}\;\times \;\mathrm{mass}\;\mathrm{of}\;\mathrm{Sample}\;\mathrm{Particles}\;\left[10\;\mathrm{mg}\right]}\right]\times \;100$$

### 2.6. Quantitation of CPDI-02 and scCPDI-02 Surface Conjugation to MP by Kexin-Mediated Ultra-Performance Liquid Chromatography (UPLC)

Average levels of CPDI-02 or scCPDI-02 conjugated to the surface of MP (*n* = 3 from at least two independent batches) were determined by kexin-mediated UPLC. CPDI-02-MP or scCPDI-02-MP [10 mg] were incubated [37 °C, 16 h] in Protease Buffer (0.1 M Tris-HCl, pH 8.5) [4 mL] containing Kex2 protease (“kexin”; SignalChem Lifesciences, Richmond, BC, Canada) [200 μg] to cleave the carboxyl side of Arg-Arg [55,56] in the Cys-Gly-Arg-Arg linker and release CPDI-02 or scCPDI-02 from PEG linkers on the MP surface. Concentrations of CPDI-02 or scCPDI-02 in MP supernatants [10,000 RCF, 10 min] were immediately determined by UPLC as described for OVA loading (Section 2.4) using a reversed-phase C-18 column (ACQUITY UPLC Peptide BEH C18 Column, Waters; 130A, 1.7 um, 2.1 × 50 mm) and pure CPDI-02 or scCPDI-02 in Protease Buffer for the standard curve. Average CPDI-02 or scCPDI-02 surface conjugation was calculated as follows:

$$\mathrm{Conjugated}\;\mathrm{peptide}\;\left(\frac{\mu g}{\mathrm{mg}}\right)=\frac{\mathrm{Conjugated}\;\mathrm{Peptide}\;\left[\frac{\mu g}{\mathrm{mL}}\right]\times \;\mathrm{Sample}\;\mathrm{Volume}\;\left[4\;\mathrm{mL}\right]}{\mathrm{Mass}\;\mathrm{of}\;\mathrm{Sample}\;\mathrm{MPs}\;\left[10\;\mathrm{mg}\right]}\times \;100$$

### 2.7. Diameters and Zeta Potentials of Microparticles

Average hydrodynamic diameters, PDI, and zeta-potentials (mV) ± SD (*n* = 3 independent samples from at least two batches) were determined using a ZetaSizer Nano ZS90 (Malvern Instruments, Malvern, UK) equipped with a He-Ne laser (λ = 633 nm) as the incident beam. Lyophilized MP were suspended in solution (10 mM NaCl in deionized H_2_O) [0.5 mg/mL] and incubated within the instrument (25 °C, 4 min) before measuring.

### 2.8. Animals

All animal procedures were approved by the University of Nebraska Medical Center Institutional Animal Care and Use Committee. Naïve female mice (C57BL/6NCrl ~8 weeks old, Charles River Laboratories, Wilmington, MA, USA) were acclimatized in an ABSL-2 facility under pathogen-free conditions at least one week before experiments.

### 2.9. Intranasal Administration

Vehicle alone (sterile PBS) [50 µL] or vehicle containing LPS-free OVA encapsulated in the indicated MP [50 µg LPS-free OVA from ~806 μg to ~961 µg MP total] at the indicated intranasal administration volume (IAV) was administered to sedated, supine mice (isoflurane-anesthetic chamber, oxygen flow 1.5 L/min, vaporized isoflurane at 2.5%, 5 min) on days −14, −7, and 0 by alternating drops between the nares using a 20 μL pipette [10 μL IAV] or 200 µL pipette [50 μL IAV]. An IAV of 10 μL is expected to deposit MP primarily in the nasal cavity of mice (i.e., intranasal immunization), whereas an IAV of 50 μL is expected to deposit MP in the nasal cavity and lungs (i.e., respiratory immunization) [25,57,58,59].

### 2.10. Isolation of Murine Lung Lymphocytes and Splenocytes 

Mice were euthanized by isoflurane overdose followed by cervical dislocation at the indicated time point after the final treatment. Following euthanasia, lungs were surgically exposed, perfused by injecting PBS [5 mL] with a 25G needle into the right ventricle of the heart, removed, minced using sterile surgical scissors, and placed directly into a sterile gentleMACS C-Tube (Miltenyi) containing collagenase IV [1 mg/mL] in 5 mL complete RPMI (cRPMI: RPMI-1640 (Hyclone), HI-FBS (Atlanta Biologicals) [10% *v*/*v*], L-glutamine (GIBCO) [2 mM], sodium pyruvate (Gibco) [1 mM], non-essential amino acids (Hyclone) [0.1 mM], MEM vitamin solution (Hyclone) [1X], penicillin G/streptomycin sulfate (Gibco) [100 U/mL/100 μg/mL], β-mercaptoethanol (Sigma) [50 μM]). Lung fragments were then homogenized with a gentleMACS Tissue Dissociator (Miltenyi) using the “m_lung_02” setting and incubated [37 °C, 30 min] in a shaking incubator (Vortem) [200 RPM]. Following incubation, digested lung fragments were homogenized again with the Tissue Dissociator using the same settings. Splenocytes were isolated using sterile forceps, minced with sterile surgical scissors, and then placed in a sterile gentleMACS C tube containing cRPMI [3 mL]. Spleens were then homogenized with a gentleMACS Tissue Dissociator using the “m_spleen_01” setting. Digested spleen or lung cell suspensions were then passed through sterile 30 μm pre-separation filters (Miltenyi) and carefully overlayed onto Lympholyte M (Cedarlane Labs) [5 mL] in a sterile 15-mL centrifuge tube. Cells were centrifuged [1500 RCF, 20 min, r.t., no brakes] and lung lymphocytes or splenocytes were collected from the interphase with a sterile Pasteur pipette and transferred to sterile 15-mL centrifuge tubes. Purified cells were then diluted to 10 mL with cRPMI, pelleted [800 RCF, r.t., 10 min], resuspended in cRPMI [7 mL], pelleted [800 RCF, r.t., 10 min], and resuspended in 0.6 mL (lung lymphocytes) or 1.2 mL (splenocytes) of serum-free CTL-Test Media (Cellular Technology Limited), counted (Cellometer Auto T4 Automated Cell Counter), and diluted in serum-free CTL-Test Media as needed for ELISpot assays (Section 2.11).

### 2.11. IgA, IgG, and IgM ELISpot Assays

Antibody-secreting cells (ASC) were quantitated using Murine Single-Color ELISpot kits (ImmunoSpot) as directed with slight modification. ELISpot plates were coated with OVA [75 μg/mL, 80 μL/well] and incubated at 4 °C overnight the day before lymphocyte/splenocyte isolations. Purified lung lymphocytes or splenocytes (Section 2.10) were diluted as needed in serum-free CTL-Test Media [5 × 10^6^ cells/mL], plated in triplicate [0.1 mL/well], and incubated at 37 °C, 5% CO_2_ for 18 h. OVA-specific ASC spots were counted with an ImmunoSpot S6 MACRO Plate Analyzer (Cellular Technology Limited) using the “Smart Count Wizard” function (B-cell specific mode; “Small Spots” setting; Spot Separation = 1; Background Balance = 10) and manually adjusting the positive spot gating threshold to a minimum surface area of 0.0015 mm^2^ (IgA and IgG) or 0.0035 mm^2^ (IgM). Average spot counts per sample were then normalized to 1 × 10^6^ plated lymphocytes or splenocytes.

### 2.12. Collection of Serum, BALF, and NLF from Mice

Serum was isolated by collecting whole blood [0.2 to 0.3 mL] into a sterile 0.5-mL centrifuge tube from a submandibular venipuncture (5-mm lancet, MEDIpoint) [60]. Blood was allowed to clot at r.t. for 30 min then centrifuged [2000 RCF, 4 °C, 10 min]. Serum was then transferred to a new sterile 0.5-mL centrifuge tube and stored at −80 °C. Bronchoalveolar lavage fluid (BALF) and nasal lavage fluid (NLF) were collected after isoflurane overdose on Day 14 and Day 90 [61,62]. The tracheas of immunized mice were surgically exposed, and an incision was made below the larynx. A cannula was then inserted and sterile PBS [1.0 mL] was instilled into the lungs and recovered by aspiration. For NLF collection, the cannula was reoriented in the trachea toward the cranium and guided toward the nasopharynx. Sterile PBS [0.6 mL] was then flushed through the nasal cavity and collected in a 6-well plate before being transferred to sterile 0.5-mL centrifuge tubes. BALF or NLF was then centrifuged [400 RCF, 4 °C, 10 min] and supernatants were stored at −80 °C.

### 2.13. OVA-Specific Antibody Titers of Serum, BALF, and NLF

Indirect ELISA was used to determine OVA-specific titers of IgG1, IgG2b, IgG2c, and IgG3 in isolated serum, IgA and total IgG in BALF, and IgA in NLF. Each class- or subclass-specific assay was completed as directed with a few modifications. For all ELISAs, OVA was suspended in ELISA coating buffer (Thermo Scientific) [0.1 mg/mL], transferred to a 96-well clear flat-bottom polystyrene high bind microplate (Corning) [0.1 mL/well], plates sealed in parafilm, and incubated at 4 °C overnight. Plates were washed with PBST [3 × 0.3 mL] using an automated plate washer (BioTek ELx50 Microplate Strip Washer) and blocked with 2X ELISA Assay Diluent (Invitrogen) [0.25 mL/well] for 2 h. Plates were washed again using same settings and 1X ELISA Assay Diluent [15 μL/well] was added to the plate. Serial 10-fold dilutions (6 dilutions minimum) of sera, BALF, or NLF of each treatment group in 1X ELISA Assay Diluent were plated and incubated at r.t. on a plate shaker [400 RPM] for 2 h. Plates were washed with PBST [3 × 0.3 mL] and HRP-conjugated detection antibodies for assay-specific isotypes were diluted in 1X diluent and added to each plate [0.1 mL/well]. Plates were incubated at r.t. on a plate shaker [400 RPM] for 1 h. The indicated HRP-conjugated detection antibodies were added to the wells including goat anti-Mouse IgG1, HRP (Invitrogen, 1:500 dilution); goat anti-Mouse IgG2b, HRP (Invitrogen) [1:500 dilution]; goat anti-mouse IgG2c, HRP (Invitrogen) [1:500 dilution]; goat anti-mouse IgG3, HRP (Cell Signaling Technology) [1:250 dilution]; goat anti-mouse IgG (H + L), HRP (Invitrogen) [1:500 dilution]; or goat anti-mouse IgA (Invitrogen) [1:250 dilution]. Plates were washed with same settings then TMB substrate solution (ThermoFisher Scientific) was added [0.1 mL/well] and incubated in the dark at r.t. for 20 min. Stop solution [0.16 M H_2_SO_4_ in H_2_O; 0.1 mL/well] was added and absorbance (ABS) was measured at 450 nm and 570 nm using a microplate reader (Spectramax iD3, Molecular Devices, San Jose, CA, USA). Net ABS for each well was calculated as follows:

$$\mathrm{Net}\;\mathrm{Absorbance}=O.D._{450\mathrm{nm}}-\left(O.D._{570\mathrm{nm}}+\mathrm{Average}\;O.D.{\;\mathrm{of}\;\mathrm{blank}\;\mathrm{wells}}_{\geq \;6\;\mathrm{wells}}\right)$$

Negative Net ABS was set to 0. Positive antibody titer cutoff thresholds for individual dilution factors were then calculated statistically at the α = 0.05 level [63] using average NET ABS for mice treated with vehicle only. Net ABS of mice from each treatment group was then compared to positive titer cutoff thresholds by plotting 4-parameter logistic (4PL) curves of NET ABS from individual mice (“bottom” value = 0) and plotting a 4PL curve of positive cutoff thresholds (“bottom” value ≥ 0.01, which is the margin of error of photometric accuracy for the Spectramax iD3 device). Individual titer values were interpolated as the dilution factor at the intersection of the respective 4PL curves for each mouse and the 4PL curve of the positive titer cutoff thresholds [64]. For NLF titers, calculated values were multiplied by a factor of 18.75 to adjust for the volume of the nasal cavity (average nasal cavity volume of mice is ~32 µL [65], whereas 600 µL of PBS was used for nasal lavage [600/32 = 18.75]).

### 2.14. Lung Histology

Histologic analyses were completed on lungs of mice on Day 90 as previously described [66]. Lungs were perfused lungs by injecting PBS [5 mL] with a 25G needle through the right ventricle of the heart. Whole lungs were removed and inflated to 10 cm H_2_O pressure with a solution of 10% formalin to preserve anatomical structure. Fixed lungs were embedded in paraffin and sections (4–5 µm) were cut and stained with hematoxylin and eosin by the University of Nebraska Medical Center Tissue Sciences Facility (Omaha, NE, USA). Sample sections were then assessed for signs of inflammation by blinded grading using a scaling system for signs of inflammation where 1 = normal, 2 = mild inflammation, 3 = moderate inflammation, 4 = obvious inflammation, and 5 = severe inflammation.

### 2.15. Statistical Analyses

All statistical analyses were performed using GraphPad Prism version 9.4.0 (San Diego, CA, USA) for Windows, www.graphpad.com (accessed on 26 July 2022). Sample outliers in all experiments were identified by the ROUT method (Q = 1%) and omitted for statistical comparisons. Data from two treatment groups were compared by two-tailed, nonparametric Mann-Whitney U Test (α = 0.05) and data from three or more treatment groups were compared by nonparametric Kruskal–Wallis one-way ANOVA with uncorrected Dunn’s multiple comparisons post hoc test (α = 0.05). Additional relevant statistical information is provided in the figure legends.

## 3. Results

### 3.1. Encapsulation of LPS-Free OVA in ~1 μm Biodegradable Microparticles Surface-Modified with CPDI-02 or Inactive, Scrambled CPDI-02

We previously encapsulated LPS-free OVA in PLGA 50:50 nanoparticles surface-modified with CPDI-02 that were ~380 nm in diameter [3]. IN administration of BSA encapsulated in PLGA 50:50 microparticles ~1 μm in diameter, however, generates higher BSA-specific titers of serum IgG in mice than BSA encapsulated in PLGA 50:50 particles ~200 nm or ~500 nm in diameter [67]. As such, we encapsulated LPS-free ovalbumin (OVA) in ~1 μm PLGA 50:50 microparticles surface-modified with CPDI-02 as a model respiratory protein vaccine.

**Figure 1:**
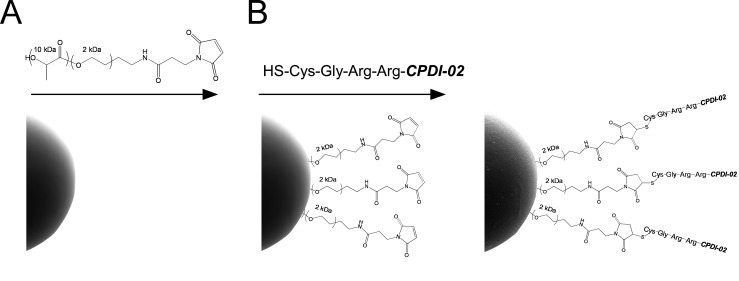
Synthetic strategy for surface modification of ~1 μm biodegradable microparticles with CPDI-02. PLGA 50:50 microparticles (MP) were (**A**) surface-activated with maleimide through 2 kDa PEG linkers by interfacial activity assisted surface functionalization (IAASF) with PLLA [10 kDa]-b-PEG [2 kDa]-maleimide diblock copolymers during encapsulation of LPS-free OVA by the W/O/W emulsification solvent extraction method (ESE), then lyophilized. (**B**) CPDI-02 was activated with sulfhydryl groups by the addition of an N-terminal Cys through a protease-labile Gly-Arg-Arg-linker then reacted with the surfaces of maleimide-activated MP resuspended in PBS and lyophilized again. Adapted from [3].

To encapsulate LPS-free ovalbumin (OVA) in biodegradable microparticles (MP) surface-modified with CPDI-02 or inactive scrambled CPDI-02 (scCPDI-02), we activated the surface of MP with maleimide (MAL) groups through 2 kDa PEG linkers by physically incorporating diblock copolymers of PLLA(10K)-PEG(2K)-MAL into PLGA 50:50 MP by interfacial activity assisted surface functionalization (IAASF) during W/O/W encapsulation of LPS-free OVA at 10 wt% theoretical loading [68,69] (Figure 1A). We then conjugated CPDI-02 or scCPDI-02 to MAL-activated PEG on the MP surface through protease-labile N-terminal Cys-Gly-Arg-Arg linkers (Figure 1B). OVA was consistently encapsulated in ~1 μm electronegative MP at 5 to 6 wt% with minimal burst release (0.4 to 0.8%) and CGRR-CPDI-02 (CPDI-02-MP) or CGRR-scCPDI-02 (scCPDI-02-MP) was conjugated to the MP surface at ~0.4 wt% (Table 1).

### 3.2. Surface Modification of ~1 μm Biodegradable Microparticles with CPDI-02 and Increased Pulmonary Delivery Increase the Magnitudes of Short-Term IgA and IgM Antibody-Secreting Cells (ASCs) against Encapsulated Protein Antigen in the Lungs of Young, Naïve Mice 

**Figure 2:**
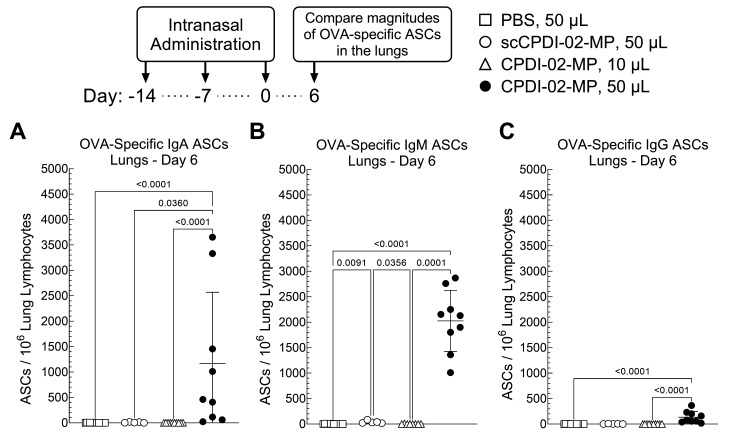
Effects of modifying the surface of ~1 μm biodegradable microparticles with CPDI-02 and increased pulmonary delivery on magnitudes of short-term antibody-secreting cells (ASCs) against encapsulated LPS-free OVA in the lungs of young, naïve female C57BL/6 mice after intranasal administration. Vehicle alone (sterile PBS [50 µL; white squares]) or vehicle containing an equivalent dose of LPS-free OVA [50 µg] encapsulated in PLGA 50:50 microparticles (~1 µm diam.) modified with surface-conjugated inactive, scrambled CPDI-02 (scCPDI-02-MP in 50 µL; white circles) or surface-conjugated CPDI-02 (CPDI-02-MP in 10 µL; white triangles or CPDI-02-MP in 50 µL; black circles) at 0.4 wt% (Table 1) was intranasally administered to ~8-week old naive female C57BL/6 mice (*n* = 5 or 10 mice) on Days −14, −7, and 0. Average OVA-specific (**A**) IgA, (**B**) IgM, and (**C**) IgG antibody secreting cell (ASC) spots/10^6^ lung lymphocytes ± SD (*n* = 3 replicates per mouse) in the lungs were then determined 6 days post-treatment by ELISpot and compared by Kruskal–Wallis nonparametric one-way ANOVA with uncorrected Dunn’s post-test. Outliers identified by the ROUT method (Q = 1%) were omitted. Data combined from two independent studies.

Transient increases in the magnitudes of antigen-specific antibody-secreting cells (ASCs) shortly after acute infection or immunization potentially correlate with subsequent long-term antibody titers [70,71]. Thus, to provide an early indication that surface modification of ~1 μm biodegradable microparticles with CPDI-02 is likely to increase titers of long-term mucosal antibodies against encapsulated protein antigen, we intranasally administered vehicle alone (PBS) or vehicle containing an equivalent dose of LPS-free OVA [50 µg] encapsulated in inactive scCPDI-02-MP or CPDI-02-MP (Table 1) to young, naïve female C57BL/6 mice once every 7 days over 14 days in an intranasal administration volume (IAV) of 50 µL that is expected to deliver microparticles to the nasal cavity and lungs (i.e., respiratory immunization) [59] (Figure 2). Given that IN administration of tetanus toxoid (TT) encapsulated in poly-L-lactide (PLA) microparticles (~1.8 μm diameter) using an IAV of 10 μL expected to deliver microparticles primarily to the nasal cavity of mice (i.e., intranasal immunization) [59] generates lower titers of anti-TT IgA and IgG in BALF than an IAV of 50 μL in BALB/mice [57], CPDI-02-MP were also administered in an IAV of 10 µL to determine possible effects of intranasal vs. respiratory immunization on mucosal and systemic antibody titers. We then compared magnitudes of short-term OVA-specific IgA, IgG, and IgM ASCs in the lungs at 6 days post-treatment (20 days post-prime) by ELISpot (Figure 2).

CPDI-02-MP in 50 μL IAV (Figure 2, black circles) increased the magnitudes of OVA-specific IgA ASCs (10^2.8^-fold) (Figure 2A), IgM ASCs (10^3^-fold) (Figure 2B), and IgG ASCs (10^2.4^-fold) (Figure 2C) in the lungs compared to 50 μL IAV alone (Figure 2, white squares). CPDI-02-MP (Figure 2, black circles) also increased magnitudes of OVA-specific IgA ASCs (144-fold) (Figure 2A) and IgM ASCs (52-fold) (Figure 2B) with a trending, but statistically insignificant, increase in OVA-specific IgG ASCs (30-fold) (Figure 2C) in the lungs compared to inactive scCPDI-02-MP in the same 50 μL IAV (Figure 2, white circles). In contrast, CPDI-02-MP in 10 μL IAV (Figure 2, white triangles) generated similar levels of OVA-specific IgA ASCs (Figure 2A), IgM ASCs (Figure 2B), and IgG ASCs (Figure 2C) in the lungs as 50 μL of the vehicle alone (Figure 2, white squares). Thus, given that a large proportion of IgM memory B-cells are expected to class-switch to IgG memory B-cells [72], surface modification of ~1 μm biodegradable microparticles with CPDI-02 and increasing delivery to the lungs will likely increase long-term mucosal antibodies against encapsulated protein antigen in young, naïve mice.

### 3.3. Surface Modification of ~1 μm Biodegradable Microparticles with CPDI-02 and Increased Pulmonary Delivery Greatly Increase Titers of Short-Term and Long-Term Mucosal Antibodies against Encapsulated Protein Antigen in the Nasal Cavity and Lungs of Young, Naïve Mice

**Figure 3:**
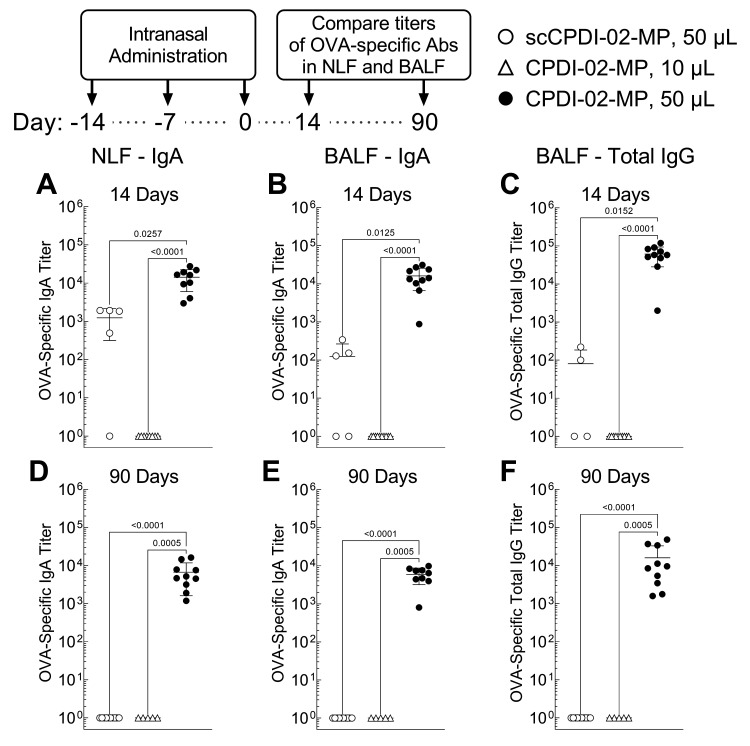
Surface modification of ~1 μm biodegradable microparticles with CPDI-02 and increased pulmonary delivery increase titers of short-term and long-term antibodies against encapsulated LPS-free OVA in the nasal cavities and lungs of young, naïve female C57BL/6 mice after intranasal administration. Vehicle alone (used for normalization) or vehicle containing an equivalent dose of LPS-free OVA [50 µg] encapsulated in PLGA 50:50 microparticles (~1 µm diam.) surface-modified with inactive scrambled scCPDI-02 (scCPDI-02-MP in 50 µL; white circles) or CPDI-02 (CPDI-02-MP in 10 µL; white triangles or CPDI-02-MP in 50 µL; black circles) through 2 kDa PEG linkers at 0.4 wt% (Table 1) was intranasally administered to ~8-week old naive female C57BL/6 mice (*n* = 5 or 10 mice) on Days −14, −7, and 0. Average OVA-specific titers ± SD (*n* = 3 replicates per mouse) of IgA in the nasal lavage fluid (NLF) (**A**,**D**) and bronchoalveolar lavage fluid (BALF) (**B**,**E**) and total IgG in the BALF (**C**,**F**) 14 days (**A**–**C**) and 90 days (**D**–**F**) post-treatment were determined by ELISA, normalized to vehicle alone by statistical endpoint titer analysis, and compared by Kruskal–Wallis nonparametric one-way ANOVA with uncorrected Dunn’s post-test. Outliers identified by the ROUT method (Q = 1%) were omitted. Titers below the positive titer cutoff threshold are shown as 10^0^. Data combined from two independent studies.

Given that CPDI-02-MP in 50 μL IAV generated higher magnitudes of IgA and IgM ASCs against encapsulated LPS-free OVA in the lungs of mice than inactive scCPDI-02-MP in 50 μL IAV 6 days post-immunization (20 days post-prime) (Figure 2), we expected CPDI-02-MP would generate higher subsequent titers of OVA-specific mucosal antibodies. To first determine if surface modification of ~1 μm biodegradable microparticles with CPDI-02 increases short-term mucosal antibodies against encapsulated protein antigen, we intranasally administered vehicle alone (50 μL), inactive scCPDI-02-MP in 50 μL IAV, or CPDI-02-MP in 10 μL or 50 μL IAV as before but compared titers of OVA-specific IgA in nasal lavage fluid (NLF) and titers of OVA-specific IgA and IgG in bronchoalveolar lavage fluid (BALF) normalized to vehicle alone 14 days post-immunization (28 days post-prime) by ELISA (Figure 3A–C). 

CPDI-02-MP in 50 μL IAV (Figure 3, black circles) increased titers of OVA-specific IgA in the NLF (11-fold) (Figure 3A) and BALF (10^2.1^-fold) (Figure 3B) and OVA-specific IgG in the BALF (10^2.8^-fold) (Figure 3C) 14 days post-treatment compared to inactive scCPDI-02-MP in the same 50 μL IAV (Figure 3A–C, white circles). CPDI-02-MP in 50 μL IAV (Figure 3, black circles) also increased titers of OVA-specific IgA in the NLF (10^4^-fold) (Figure 3A) and BALF (10^4.2^-fold) (Figure 3B) and OVA-specific IgG in the BALF (10^4.8^-fold) (Figure 3C) 14 days post-treatment compared to CPDI-02-MP in 10 μL IAV (Figure 3A–C, white triangles). Thus, surface modification of ~1 μm biodegradable microparticles with CPDI-02 and increasing delivery to the lungs significantly increases short-term mucosal antibodies against encapsulated protein antigen in young, naïve mice.

To next determine if surface modification of biodegradable microparticles with CPDI-02 increases the generation of long-term mucosal antibodies against encapsulated protein antigen, we intranasally administered vehicle alone (50 μL), inactive scCPDI-02-MP in 50 μL IAV, or CPDI-02-MP in 10 μL or 50 μL IAV as before (Figure 2) and compared titers of OVA-specific IgA in NLF and titers of OVA-specific IgA and IgG in BALF normalized to vehicle alone 90 days post-immunization (104 days post-prime) by ELISA (Figure 3D–F).

CPDI-02-MP in 50 μL IAV (Figure 3, black circles) increased titers of OVA-specific IgA in the NLF (10^3.8^-fold) (Figure 3D) and BALF (10^3.8^-fold) (Figure 3E) and titers of OVA-specific IgG in the BALF (10^4.2^-fold) (Figure 3F) 90 days post-treatment compared to undetectable titers from inactive scCPDI-02-MP in the same 50 μL IAV (Figure 3D–F, white triangles). CPDI-02-MP in 50 μL IAV (Figure 3, black circles) also increased titers of OVA-specific IgA in the NLF (10^3^-fold) (Figure 3D) and BALF (10^4^-fold) (Figure 3E) and titers of OVA-specific IgG in the BALF (10^4.4^-fold) (Figure 3F) 90 days post-immunization compared to undetectable titers from CPDI-02-MP in 10 μL IAV (Figure 3D–F, white triangles). Thus, surface modification of ~1 μm biodegradable microparticles with CPDI-02 and increasing delivery to the lungs significantly increases long-term mucosal antibodies against encapsulated protein antigen in young, naïve mice.

### 3.4. Effect of Surface Modification of ~1 μm Biodegradable Microparticles with CPDI-02 and Increased Pulmonary Delivery on Magnitudes of Short-Term Systemic Antibody-Secreting Cells (ASCs) against Encapsulated Protein Antigen in Young, Naïve Mice

**Figure 4:**
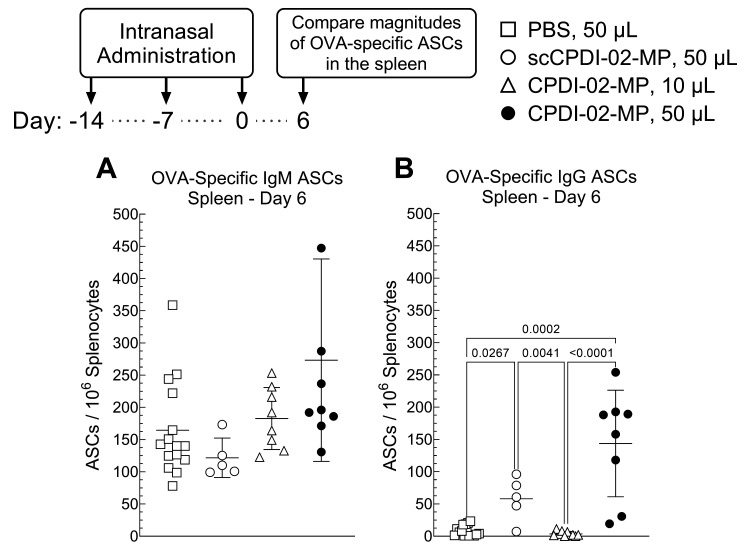
Effects of modifying the surface of ~1 μm biodegradable microparticles with CPDI-02 and increased pulmonary delivery on magnitudes of short-term systemic OVA-specific antibody-secreting cells (ASCs) against encapsulated LPS-free OVA in naïve female C57BL/6 mice after intranasal administration. Vehicle alone (PBS, 50 µL) or vehicle containing an equivalent dose of LPS-free OVA [50 µg] encapsulated in PLGA 50:50 microparticles (~1 µm diam.) surface-modified with 0.4 wt% inactive, scrambled scCPDI-02 (scCPDI-02-MP, 50 µL) or CPDI-02 (CPDI-02-MP, 10, or 50 µL) through 2 kDa PEG linkers was intranasally administered to naive female C57BL/6 mice (*n* = 5 to 15 mice) on Days −14, −7, and 0. Average OVA-specific (**A**) IgM or (**B**) IgG antibody secreting cell (ASC) spots/10^6^ splenocytes ± SD (*n* = 3 replicates per mouse) were determined 6 days post-treatment by ELISpot and compared by Kruskal–Wallis nonparametric one-way ANOVA with uncorrected Dunn’s post-test. Outliers identified by the ROUT method (Q = 1%) were omitted. Data combined from three independent studies.

Vaccine administration to the respiratory tract, such as other routes of mucosal administration [73], potentially generates both mucosal and systemic antibodies [74]. To provide an initial indication that surface modification of ~1 μm biodegradable microparticles with CPDI-02 and increasing delivery to the lungs will likely increase systemic antibodies against encapsulated protein antigen, we intranasally administered vehicle alone (50 μL), inactive scCPDI-02-MP in 50 μL IAV, CPDI-02-MP in 10 μL IAV, or CPDI-02-MP in 50 μL IAV as before but compared magnitudes of OVA-specific IgM and IgG ASCs in the spleen 6 days post-treatment (20 days post-prime) by ELISpot (Figure 4). We focused on systemic IgM given that IgM memory B-cells are most abundant in the early stages of immunization or infection and undergo T helper cell (Th)-dependent class-switching to IgG memory B-cells and on systemic IgG ASCs given that the highest proportion of circulating antibodies are IgG [72].

CPDI-02-MP in 50 μL IAV (Figure 4A, black circles) generated similar levels of OVA-specific IgM ASCs in the spleen as 50 μL of vehicle alone (Figure 4A, white squares), inactive scCPDI-02-MP in 50 μL IAV (Figure 4A, white circles), and CPDI-02-MP in 10 μL IAV (Figure 4A, white triangles). In contrast, CPDI-02-MP in 50 μL IAV (Figure 4B, black circles) generated 19-fold higher levels of OVA-specific IgG ASCs than vehicle alone (Figure 4B, white squares), 41-fold higher levels of OVA-specific IgG ASCs than CPDI-02-MP in 10 μL IAV (Figure 4B, white triangles), but similar levels of OVA-specific IgG ASCs in the spleen as inactive scCPDI-02-MP in the same 50 μL IAV (Figure 4B, white circles). Thus, differences between magnitudes of ASCs in the spleens of young, naïve mice 6 days post-intranasal immunization (20 days post-prime) suggest that increasing ~1 μm microparticle delivery to the lungs likely increases systemic antibodies against encapsulated protein antigen but the effects of modifying the microparticle surface with CPDI-02 remained unclear under the current experimental conditions.

### 3.5. Surface Modification of ~1 μm Biodegradable Microparticles with CPDI-02 and Increased Pulmonary Delivery Greatly Increase Short-Term and Long-Term Systemic IgG Antibody Subclasses against Encapsulated Protein Antigen in the Sera of Young, Naïve Mice

Given that CPDI-02-MP in 50 μL IAV generated higher magnitudes of systemic IgG ASCs against encapsulated LPS-free OVA than in 10 μL IAV but similar magnitudes as inactive scCPDI-02-MP in 50 μL IAV (Figure 4), it remained unclear if both surface modification of ~1 μm biodegradable microparticles with CPDI-02 and increased delivery to the lungs are likely to increase systemic antibodies against encapsulated protein antigen as observed for antibodies in the nasal cavities and lungs (Figure 3). 

**Figure 5:**
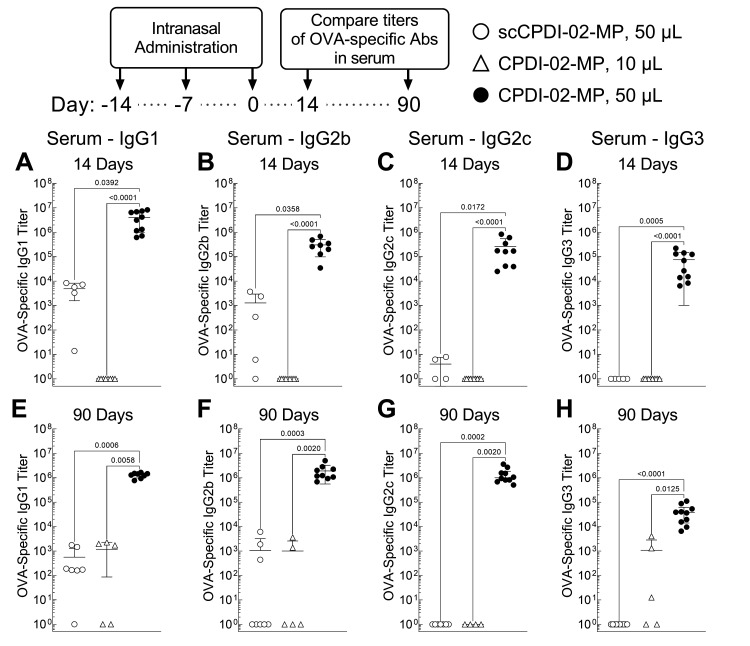
Surface modification of biodegradable microparticles with CPDI-02 and increased pulmonary delivery increase titers of short-term and long-term systemic IgG subclasses against encapsulated LPS-free OVA in naïve female C57BL/6 mice after intranasal administration. Vehicle alone (used for normalization) or vehicle containing an equivalent dose of LPS-free OVA [50 µg] encapsulated in PLGA 50:50 microparticles (~1 µm diam.) surface-modified with inactive scrambled CPDI-02 (scCPDI-02-MP [50 µL]; white circles) or CPDI-02 (CPDI-02-MP in 10 µL; white triangles or CPDI-02-MP in 50 µL; black circles) through 2 kDa PEG linkers at 0.4 wt% (Table 1) was intranasally administered to ~8-week old naive female C57BL/6 mice (*n* = 5 or 10 mice) on Days −14, −7, and 0. Average OVA-specific titers ± SD (*n* = 3 replicates per mouse) of Th2 IgG1 antibodies (**A**,**E**) and Th1 IgG2b (**B**,**F**), IgG2c (**C**,**G**), and IgG3 (**D**,**H**) antibodies in the serum were determined 14 days (**A**–**D**) and 90 days (**E**–**H**) post-treatment by ELISA, normalized to vehicle alone by statistical endpoint titer analysis, and compared by Kruskal–Wallis nonparametric one-way ANOVA with uncorrected Dunn’s post-test. Outliers identified by the ROUT method (Q = 1%) were omitted. Titers below the positive titer cutoff threshold are shown as 10^0^. Data combined from two independent studies.

To first determine if conjugating CPDI-02 to the surface of ~1 μm biodegradable microparticles and increasing delivery to the lungs increases short-term systemic IgG antibodies against encapsulated protein antigen, we intranasally administered vehicle alone (50 μL), inactive scCPDI-02-MP in 50 μL IAV, CPDI-02-MP in 10 μL IAV, or CPDI-02-MP in 50 μL IAV as before but compared serum titers of OVA-specific IgG subclasses normalized to vehicle alone 14 days post-treatment (28 days post-prime) by ELISA (Figure 5A–D). We compared effects on each IgG subclass given that, according to the quartet model of murine IgG function [75], murine IgG subclasses likely work together to clear infections as follows: Th-independent IgG3 and IgG2b ensure activation of inflammation and FcγR-mediated effector functions (IgG2b only) during the early stages of infection, whereas Th-dependent IgG2a (IgG2c in C57BL/6 mice) and IgG1 increase pathogen clearance and limit IgG-driven inflammation, respectively, during the later stages of infection.

CPDI-02-MP in 50 μL IAV (Figure 5, black circles) generated much higher serum titers of OVA-specific IgG1 (10^3^-fold) (Figure 5A), IgG2b (10^2.4^-fold) (Figure 5B), IgG2c (10^5^-fold) (Figure 5C), and IgG3 (10^5^-fold) (Figure 5D) 14 days post-treatment than inactive scCPDI-02-MP in the same 50 μL IAV (Figure 5A–D, white circles). CPDI-02-MP in 50 μL IAV (Figure 5, black circles) also generated much higher serum titers of OVA-specific IgG1 (10^6.6^-fold) (Figure 5A), IgG2b (10^5.5^-fold) (Figure 5B), IgG2c (10^5.4^-fold) (Figure 5C), and IgG3 (10^5^-fold) (Figure 5D) 14 days post-treatment than CPDI-02-MP in 10 μL IAV (Figure 5A–D, white triangles). Thus, surface modification of ~1 μm biodegradable microparticles with CPDI-02 and increasing delivery to the lungs significantly increases short-term systemic IgG antibody subclasses against encapsulated protein antigen in young, naïve mice.

To next determine if surface modification of ~1 μm biodegradable microparticles with CPDI-02 and increasing pulmonary delivery increases long-term systemic antibodies against encapsulated protein antigen, we intranasally administered vehicle alone (50 μL), inactive scCPDI-02-MP in 50 μL IAV, CPDI-02-MP in 10 μL IAV, or CPDI-02-MP in 50 μL IAV as before but compared serum titers of OVA-specific IgG subclasses normalized to vehicle alone 90 days post-treatment by ELISA (Figure 5E–H). Similar to 14 days post-immunization (28 days post-prime)(Figure 5A–D), CPDI-02-MP in 50 μL IAV (Figure 5, black circles) generated much higher serum titers of OVA-specific IgG1 (10^3.4^-fold) (Figure 5E), IgG2b (10^3.3^-fold) (Figure 5F), IgG2c (10^6.1^-fold) (Figure 5G), and IgG3 (10^4.6^-fold) (Figure 5H) 90 days post-treatment than inactive scCPDI-02-MP in the same 50 μL IAV (Figure 5E–H, white circles). 

CPDI-02-MP in 50 μL IAV (Figure 5, black circles) also generated much higher serum titers of OVA-specific IgG1 (10^3.1^-fold) (Figure 5E), IgG2b (10^3.3^-fold) (Figure 5F), IgG2c (10^6.1^-fold) (Figure 5G), and IgG3 (10^1.6^-fold) (Figure 5H) 90 days post-treatment than CPDI-02-MP in 10 μL IAV (Figure 5E–H, white triangles). Thus, surface modification of ~1 μm biodegradable microparticles with CPDI-02 and increasing delivery to the lungs significantly increases long-term systemic IgG antibody subclasses against encapsulated protein antigen in young, naïve mice.

### 3.6. Preliminary Assessment of Long-Term Lung Inflammation in Healthy Young Mice after Respiratory Immunization with Surface-Modified MP

**Figure 6:**
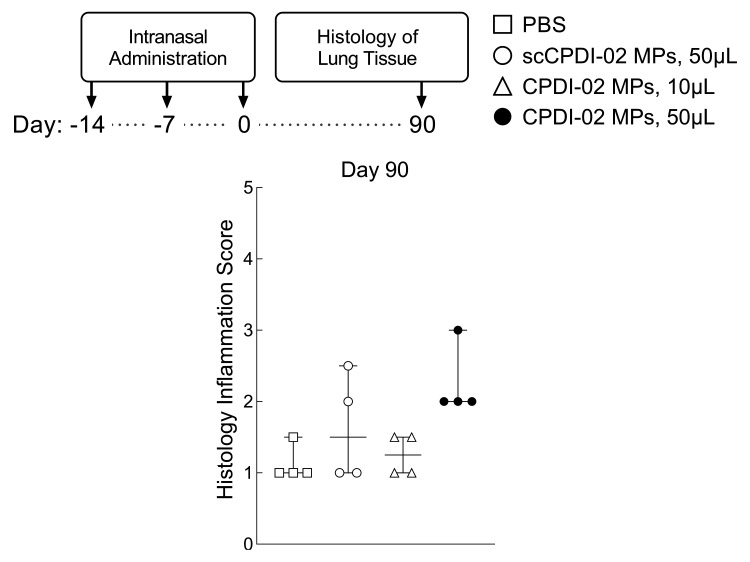
Comparison of long-term lung inflammation in naïve female C57Bl/6 mice after IN administration of vehicle, inactive scCPDI-02-MP, or CPDI-02-MP. Vehicle alone (PBS [50 µL]; white squares) or vehicle containing an equivalent dose of LPS-free OVA [50 µg] encapsulated in PLGA 50:50 microparticles (~1 µm diam.) surface-modified with inactive scrambled CPDI-02 (scCPDI-02-MP [50 µL]; white circles) or CPDI-02 (CPDI-02-MP in 10 µL, white triangles or CPDI-02-MP in 50 µL, black circles) through 2 kDa PEG linkers at 0.4 wt% (Table 1) was intranasally administered to ~8-week old naive female C57BL/6 mice on Days −14, −7, and 0 (Figure 3 and Figure 5). On Day 90 (104 days post-prime), median scores of lung inflammation (single-blind) ± range (*n* = 4 mice) were determined by blinded histology grading (1 = normal, 2 = mild inflammation, 3 = moderate inflammation, 4 = obvious inflammation, and 5 = severe inflammation) and compared by nonparametric Kruskal–Wallis one-way ANOVA with uncorrected Dunn’s test.

To provide a preliminary assessment of long-term lung inflammation after respiratory immunization with CPDI-02-MP, we intranasally administered vehicle alone (50 μL), inactive scCPDI-02-MP in 50 μL IAV, CPDI-02-MP in 10 μL, or CPDI-02-MP in 50 μL IAV as before, but compared the extent of lung inflammation 90 days post-immunization (104 days post-prime) by blinded histology grading (Figure 6). CPDI-02-MP in 50 μL IAV (Figure 6, black circles) had similar scores of normal to mild inflammation as vehicle alone (Figure 6, white squares), inactive scCPDI-02-MP (Figure 6, white circles), and CPDI-02-MP in 10 μL IAV (Figure 6, white triangles). Thus, respiratory immunization with ~1 μm CPDI-02-MP causes mild long-term inflammation in the lungs of healthy young mice under the current experimental conditions.

## 4. Discussion

Our study provides evidence that surface modification of ~1 μm biodegradable microparticles with CPDI-02 significantly increases long-term mucosal and systemic antibodies against encapsulated protein antigen in young naïve mice after respiratory immunization with minimal long-term inflammation in the lungs. We found that IN administration of LPS-free OVA protein encapsulated in ~1 μm PLGA 50:50 MP surface-modified with 0.4 wt% CPDI-02 (CPDI-02-MP) through 2 kDa PEG linkers (Table 1) to naïve female C57BL/6 mice in 50 μL of vehicle (“respiratory immunization”) greatly increased titers of IgA in NLF and BALF (Figure 2), total IgG in BALF (Figure 2), and serum titers of IgG1, IgG2b, IgG2c, and IgG3 (Figure 5) against encapsulated LPS-free OVA and showed similar signs of mild inflammation in the lungs 90 days post-immunization (104 days post-prime) (Figure 6) compared to LPS-free OVA protein encapsulated in MP surface-modified with 0.4 wt% inactive scrambled scCPDI-02.

Our study also provides evidence that increasing delivery of ~1 μm microparticles to the lungs increases long-term mucosal and systemic antibodies against encapsulated protein antigen in young naïve mice. We found that IN administration of CPDI-02-MP (Table 1) to naïve female C57BL/6 mice in an IAV of 50 μL expected to deposit ~1 μm microparticles primarily in the nasal cavity and lungs of mice (“respiratory immunization”) [59] greatly increased titers of OVA-specific IgA in NLF and BALF (Figure 2), total OVA-specific IgG in BALF (Figure 2), and serum titers of OVA-specific IgG1, IgG2b, IgG2c, and IgG3 (Figure 5) 90 days post-immunization (104 days post-prime) compared to CPDI-02-MP in an IAV of 10 μL expected to deposit microparticles in the nasal cavity alone [59]. Our findings are similar to a previous report that increasing the IAV from 10 μL to 50 μL increases titers of IgA and IgG in the BALF of BALB/c mice against tetanus toxoid encapsulated in ~1.8 μm PLA microparticles [57]. This suggests that nasal-associated lymphoid tissue (NALT) in mice is not involved in generating mucosal and systemic antibodies by ~1 μm CPDI-02-MP. Considering that the volume of the nasal cavity of ~8-week old mice is ~32 μL [65], however, it remains possible that IAVs > 30 μL are required to deliver a sufficient amount of CPDI-02-MP to the NALT. Thus, distribution studies with IAVs between 10 μL and 50 μL will be required to determine the extent of NALT involvement with ~1 μm CPDI-02-MP in mice.

Surface modification of ~1 μm biodegradable microparticles with CPDI-02 may significantly increase long-term mucosal and systemic antibodies against encapsulated protein antigen by initially increasing MP localization to MALT in the nasal cavities (NALT) and lungs (BALT) through increased affinity for C5aR1 receptors expressed on the surface of microfold/membrane cells (M cells) within follicle associated epithelium (FAE) [76]. A similar mechanism has been proposed for M cell-targeted oral vaccines that use ligands to increase affinity for C5aR1 [76,77] or other M cell surface proteins [78]. Whether the nonspecific affinity of MP for M cells already maximizes the rate of M cell transcytosis into MALT or if the potential activation of C5aR1 by surfacCPDI-02 increases the rate of M-cell transcytosis also remains unclear [3]. CPDI-02-MP in the subepithelial compartment of MALT could then (i.) prolong the local release of encapsulated protein antigen for sustained activation of naïve B-cells in the MALT and MALT-draining LN (ii.) activate DC and macrophages in MALT and DC in MALT-draining LN through C5aR1 to create a cytokine microenvironment that supports the generation of long-lived memory B-cells and Th-cells (iii.) localize to MALT-draining LN as observed with 1.1 μm fluorescent polystyrene carboxylate microspheres after IN administration to mice [24] and (iv.) be phagocytosed by CPDI-02-activated DC in the MALT and MALT-draining LN for sustained antigen presentation and activation of naïve Th-cells that, in turn, support the expansion and differentiation of activated naïve B-cells into memory B-cells.

The primary limitation of our study is that it compares titers of long-term mucosal and systemic antibodies against the widely studied model protein antigen OVA but not long-term titers of antibody effector functions (e.g., neutralizing titers, ADCC titers) against a protective antigen. As such, the effect of CPDI-02 surface modification on long-term titers of mucosal and systemic antibody effector functions against encapsulated protective antigens will need to be determined for each pathogen.

## 5. Conclusions

In summary, our study indicates that surface modification of ~1 μm biodegradable microparticles with CPDI-02 through 2 kDa PEG linkers is likely to greatly increase long-lived mucosal and systemic antibodies against encapsulated protein antigen after respiratory immunization and may be an effective incorporation strategy for other routes of mucosal immunization.

## Figures and Tables

**Table 1:** Representative characteristics of LPS-free OVA encapsulated in PLGA 50:50 microparticles (MP) surface-modified with CPDI-02 or inactive scrambled scCPDI-02.

Formulation	OVA Loading ^1^ (μg/mg MP ± SD)	Burst Release ^1^ (% Loaded OVA)	CPDI-02 Conjugation ^2^ (μg/mg MP ± SD)	Diameter ^3^ (μm ± SD)	Polydispersity Index ^3^ (PDI ± SD)	Zeta Potential ^3^ (mv ± SD)
CPDI-02-MP	62 ± 13	0.8 ± 0.2	4.0 ± 0.6	1.1 ± 0.2	0.3 ± 0.1	−22 ± 3
scCPDI-02-MP	52 ± 13	0.4 ± 0.1	3.9 ± 0.2	1.21 ± 0.02	0.36 ± 0.08	−24 ± 4

^1^ OVA loading and total encapsulated OVA released 24 h after resuspension in PBS (“burst release”) determined by ultra-high performance liquid chromatography (UPLC). ^2^ Surface modification of MP with CPDI-02 or scCPDI-02 determined by kexin-mediated release/UPLC. ^3^ Diameters, polydispersity indices, and zeta potentials in 10 mM NaCl was determined by dynamic light scattering (DLS). Results are representative of at least two independent batches.

## Data Availability

Data presented in this study are available on request from the corresponding author.
